# Evaluating a Chatbot as a Companion for Patients With Breast Cancer: Collaborative Pilot Study

**DOI:** 10.2196/68426

**Published:** 2025-08-13

**Authors:** Sebastian Daniel Boie, Esther Glastetter, Michael Patrick Lux, Felix Balzer, Christof von Kalle, Christian Lenz, Ulrike Müller

**Affiliations:** 1 Pfizer Pharma GmbH, Friedrichstr. 110, Berlin, 10117, Germany, 49 15152377580; 2 Department for Gynecology and Obstetrics, St. Louise Women's Hospital, Paderborn, St. Josefs Women's Hospital, Salzkotten, St. Vincenz Clinics, Salzkotten + Paderborn, Germany; 3 Institute of Medical Informatics, Charité - Universitätsmedizin Berlin, Corporate Member of Freie Universität Berlin and Humboldt-Universität zu Berlin, Berlin, Germany; 4 Clinical Study Center, Berlin Institute of Health at Charité – Universitätsmedizin Berlin, Berlin, Germany

**Keywords:** breast cancer, chatbot, patient information, Generative artificial intelligence, Gen AI, Retrieval-augmented generation, RAG

## Abstract

**Background:**

Patients with breast cancer frequently experience significant uncertainty, prompting them to seek detailed, personalized, and reliable medical information to enhance adherence to prescribed treatments, medications, and recommended lifestyle adjustments. Although high-quality information exists within oncology guidelines and patient-oriented resources, the provision of tailored responses to individual patient queries remains challenging, especially for non–English-speaking populations.

**Objective:**

This study aims to evaluate the potential of an artificial intelligence–driven chatbot, specifically leveraging ChatGPT (GPT-4; OpenAI) combined with retrieval-augmented generation, to deliver personalized answers to complex breast cancer-related patient questions in German.

**Methods:**

We collaborated with one of Germany’s largest breast cancer Patient Representation Groups to collect authentic patient inquiries, receiving a total of 118 questions. After initial screening, we selected 104 medical questions, organized into 7 distinct categories: aftercare, bone health, ductal carcinoma in situ, diagnostics, nutrition and supplements, complementary medicine, and therapy. A customized version of GPT-4 was configured with specific system prompts emphasizing empathetic, evidence-based responses and integrated with a comprehensive database comprising guidelines, recommendations, and patient information materials published by recognized German medical societies. To assess chatbot responses, we used 4 evaluation criteria: comprehensibility (clarity from a patient perspective), correctness (accuracy per current medical guidelines), completeness (inclusion of all relevant aspects), and potential harm (risk of undue patient harm or misinformation). Ratings were conducted using a 5-point Likert scale by a breast cancer expert (correctness, completeness, and potential harm) and patient representatives (comprehensibility).

**Results:**

The chatbot provided high-quality responses across multiple dimensions. Of the 499 responses evaluated for comprehensibility, 427 (85.6%) were rated as comprehensible. Among the 104 responses assessed for the remaining dimensions, 91 (87.5%) were rated as correct, 72 (69.2%) as complete, and 93 (89.4%) as nonharmful. Reasons for incomplete answers included omission of reimbursement details, updates from recent therapeutic guidelines, or nuanced recommendations regarding endocrine therapy and aftercare schedules. In addition, 6 (5.8%) of the answers were rated as potentially harmful due to outdated or contextually inappropriate recommendations. The chatbot also performed well in the nutrition and bone health categories despite occasionally incomplete document retrieval.

**Conclusions:**

Our findings demonstrate that an artificial intelligence–powered chatbot with GPT-4 and retrieval augmentation can effectively provide personalized, linguistically accessible, and largely accurate information to German-speaking patients with breast cancer. This approach holds considerable promise for improving patient-centered communication, empowering patients to make informed decisions. Nonetheless, observed limitations regarding response completeness and potential harm underscore the critical need for ongoing human oversight. Future research and development should prioritize regularly updated databases, advanced retrieval methods to handle complex document structures, multimodal capabilities, and clearly articulated disclaimers emphasizing the necessity of professional medical consultation. Our evaluation, along with the provided set of realistic patient questions, establishes a benchmark for future development and validation of German-language oncology chatbots.

## Introduction

Breast cancer (BC) is the most frequent form of cancer in women [1,2] and a global health concern. Patients with BC have a substantial need for information on their disease at all stages of the patient journey, prefer information tailored to their individual circumstances [3-5], and it is well-known that information on disease and treatments is an important factor for medication adherence and persistence [6-9]. While health care professionals (HCPs) aim to provide comprehensive answers, time constraints and resource limitations can lead to a mismatch in information provision.

Many patients routinely use the internet as a source for information about their condition [5,10], which increases their risk of exposure to misinformation [11,12]. It is expected that patients increasingly turn to artificial intelligence (AI)–based solutions, such as chatbots, which can help to assess the credibility of information. Chatbots can play a significant role in informing patients about their situation and treatment options [13-16]. A substantial benefit of digital information sources is the easy 24-hour access to information and that these sources may answer questions that were left out during consultation by the HCPs. This may help patients have a more informed consultation with their HCP and supports shared decision-making.

Chatbot apps based on large language models (LLMs) are promising as interactive assistants to tailor medical information for patients’ specific needs [17-20]. Most existing chatbots are primarily trained on English language data, creating a language barrier for non–English-speaking patients [21,22]. LLM-based chatbots can generate conversational and personalized answers that include context. In general, medical practice differs significantly between different countries due to differences in reimbursements, regulatory frameworks, and cultural attitudes, among other factors [23,24]. While English-speaking LLM-based chatbots are developed for other cancer entities [25], there are, to the best of our knowledge, no existing publications about LLM-powered chatbot solutions for German-speaking patients with BC.

The major disadvantage of LLMs is that they can confidently generate various types of false answers (eg, hallucinations, confabulations, misrepresentations, and omissions among others). A taxonomy of false output and mitigation strategies is a topic of current research [26,27]. A mitigation strategy is to map user questions by classifying their intent to predefined answers. Solutions with predefined answers exist for lung cancer in Japanese [28] and prostate cancer in German [29]. While this approach is generally more reliable, since answers are preselected and quality controlled, it lacks flexibility and personalization of the answers.

Another emerging strategy to mitigate the risk of false information is retrieval-augmented generation (RAG) [30-32] where a trained LLM, such as a generative pretrained transformer (GPT), has access to additional information sources (eg, a database with oncology guidelines and other quality-controlled documents). This additional information can provide up-to-date and quality-controlled context for an LLM to a given query [33].

Ultimately, as AI technologies continue to evolve, health care institutions and organizations may increasingly explore the development of their own LLM-based apps to support more inclusive and patient-centered care. For such efforts to be effective and responsible, they must be grounded in language- and context-specific considerations that reflect patients’ real-world concerns.

The aim of this paper is to explore the potential of a retrieval-augmented German-language chatbot based on ChatGPT to address typical information needs of breast cancer patients using real patient questions.

## Methods

### Overview

This study was conducted in Germany as a collaborative project between researchers from industry and academic experts in digital health, oncology, and AI. An essential partner in this initiative was one of the country’s largest breast cancer Patient Representation Groups, comprising individuals with lived experience of breast cancer and deep engagement in patient advocacy. The Patient Representation Group contributed real-world patient questions and helped shape the evaluation criteria, enabling the assessment of the chatbot in a way that reflects the practical needs and concerns of breast cancer patients in the German health care context. This study was performed in accordance with the TRIPOD+LLM (Transparent Reporting of a Multivariable Prediction Model for Individual Prognosis or Diagnosis+Large Language Model) guidelines (the TRIPOD+LLM checklist is provided in Checklist 1).

**Figure 1.**
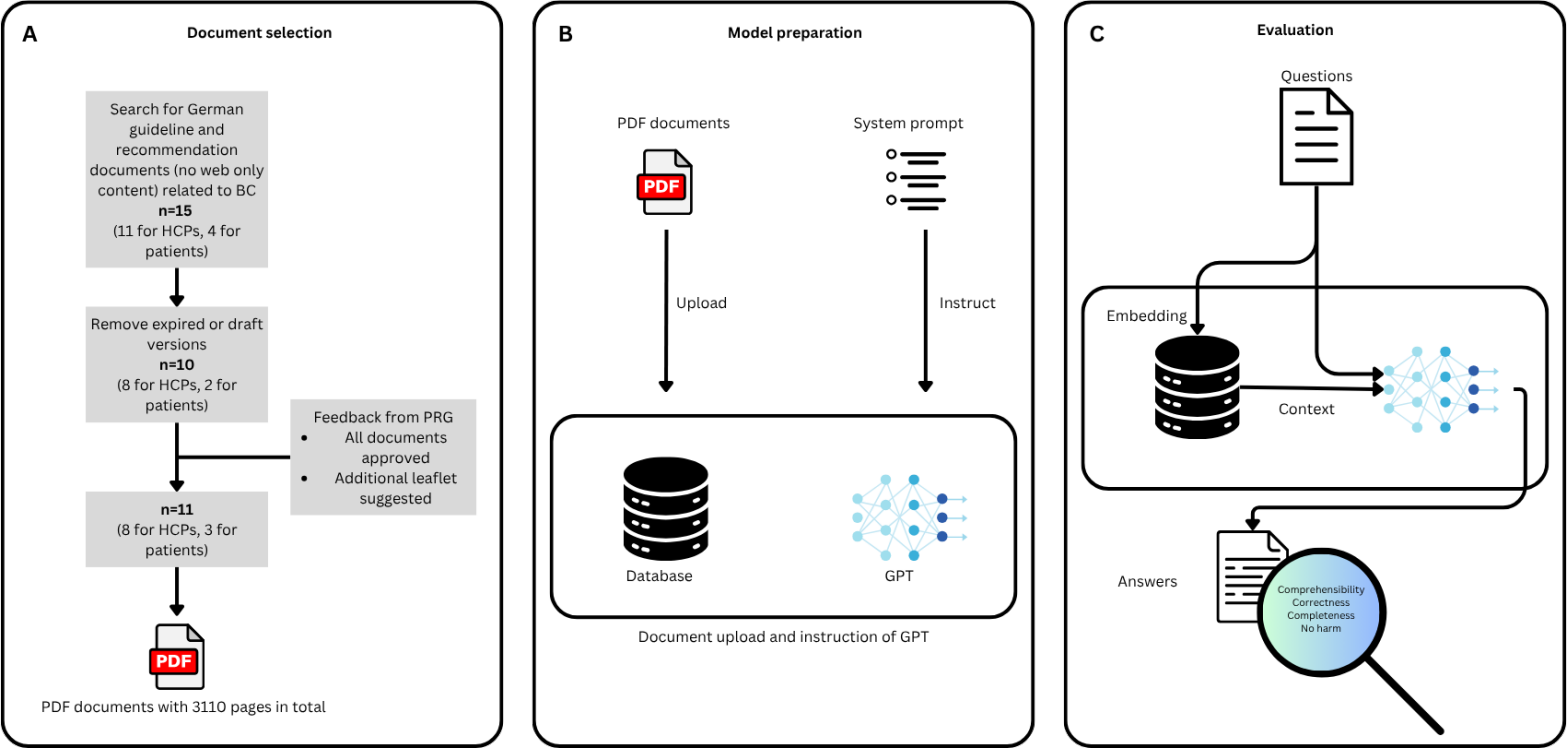
Overview of the document selection process, chatbot preparation, and evaluation. BC: Breast cancer; HCP: health care professional; PRG: Patient Representation Group.

The initial phase of the work, including chatbot refinement, question selection, and response evaluation, was completed in April 2024 (see Figure 1).

### Document Selection

We selected documents with the purpose of providing the chatbot with up-to-date, evidence-based guidelines and recommendations, ensuring that its responses are grounded in the current standard of care for BC in Germany.

Oncology guidelines and recommendations provided by professional societies are reliable, high-quality sources of information on the diagnosis and treatment of BC. The guidelines are based on expert consensus and compile evidence-based information with a comprehensive coverage and regular updates [34,35]. Guidelines are designed for clinicians to develop tailored diagnosis and treatment plans to the patients’ individual tumor biology [36].

Some societies (the German Cancer Society [DKG e.V.], the Arbeitsgemeinschaft Gynäkologische Onkologie e.V. [AGO e.V.], and the Commission Breast of the German Society of Gynecology and Obstetrics) additionally provide information sources directly for patients.

The authors screened and reviewed guidelines and information documents from prominent German medical societies, BC working groups, and nonprofit organizations providing material for HCPs and patients. The entities were selected based on their established role and recognition within the German BC clinical and patient support landscape. A consensus from all authors was reached through discussion and feedback from the Patient Representation Group solicited.

We considered documents from well-known entities (see Textbox 1).

Textbox 1. **Medical entities considered for document selection.**

We considered documents from the following well-known entities:

- Arbeitsgemeinschaft der Wissenschaftlichen Medizinischen Fachgesellschaften e.V. (AWMF).
- Deutsche Krebsgesellschaft e.V. (DKG).
- Deutschen Krebshilfe e.V. (DKH).
- Deutsche Gesellschaft für Gynäkologie und Geburtshilfe e.V. (DGGG).
- PRIO (Prävention und Integrative Onkologie, eine Arbeitsgemeinschaft der DKG).
- Deutsche Gesellschaft für Hämatologie und Medizinische Onkologie e.V. (DGHO).
- Deutschen Gesellschaft für Ernährungsmedizin e.V. (DGEM).
- Arbeitsgemeinschaft, Supportive Maßnahmen in der Onkologie, Rehabilitation und Sozialmedizin der Deutschen Krebsgesellschaft (ASORS).
- Österreichische Arbeitsgemeinschaft für klinische Ernährung (AKE).
- Dachverband der Deutschsprachigen Wissenschaftlichen Osteologischen Gesellschaften e.V. (DVO).
- Arbeitsgemeinschaft Gynäkologische Onkologie e.V. (AGO).
- Arbeitsgemeinschaft für Psychoonkologie in der Deutschen Krebsgesellschaft (PSO).

And selected all documents addressing HCPs or patients that cover diagnosis, treatment, and aftercare of breast cancer; complementary medicine for oncological patients; nutrition in clinical oncology; osteoporosis and psycho-oncological diagnosis, consultation, and treatment.

Each document was manually reviewed, and we excluded documents that are either expired or not yet consented (ie, draft versions and versions for discussion only) from our selection. Draft, discussion, or expired marks (as assigned by the Arbeitsgemeinschaft der Wissenschaftlichen Medizinischen Fachgesellschaften e.V.) were verified by 2 authors (SDB and UM). The selection was internally reviewed and discussed with representatives from the BC Patient Representation Group as well as the senior BC expert. All suggestions by one of the authors or the Patient Representation Group made it into the final selection. In total, we included 13 documents comprising 3110 pages (Figure 1A). The documents are available through the respective entities’ website, and the compendium can be used by other researchers.

The included documents are shown in Table 1.

A list of excluded documents can be found in Multimedia Appendix 1.

**Table 1.** Included documents.

Document title	Type of document	Primary target audience	Publisher or leading Medical Societies
Interdisziplinäre S3-Leitlinie für die Früherkennung, Diagnostik, Therapie und Nachsorge des Mammakarzinoms. Langversion 4.4, June 2021, Register 032-045OL	S3 Guideline	HCPs	AWMF, DKG, DKH, DGGG, and DKG.
S3-Leitlinie Komplementärmedizin in der Behandlung von onkologischen PatientInnen Langversion 1.1, September 2021, Register 032/055OL	S3 Guideline	HCPs	AWMF, DKG, DKH, DKG, PRIO, DGGG, and DGHO.
Klinische Ernährung in der Onkologie. 2015, Register 073/006. (DOI 10.1055/s-0035-1552741).	S3 Guideline	HCPs	DGEM with DGHO, ASORS, and AKE.
Prophylaxe, Diagnostik und Therapie der OSTEOPOROSE. Langfassung, September 2023, Register 183/001	S3 Guideline	HCPs	DVO.
Diagnostik und Therapie früher und fortgeschrittener Mammakarzinome 2023.1	Recommendations	HCPs	AGO Breast Commission (of DGGG) and DKG.
Mammakarzinom der Frau. January 2018	Recommendations	HCPs	Onkopedia, DGHO.
Psychoonkologische Diagnostik, Beratung und Behandlung von erwachsenen Krebspatient*innen. Version 2.1 – August 2023	S3 Guideline	HCPs	AWMF, DKG, DKH, DKG, and PSO.
Peri- und Postmenopause – Diagnostik und Interventionen. Register 015-‐062, Version 1.1, January 2020	S3 Guideline	HCPs	DGGG.
Patientinnenleitlinie. Brustkrebs im frühen Stadium. December 2018	Patient Guideline based on S3 Guideline	Patients	AWMF, DKG, and Stiftung Deutsche Krebshilfe.
BRUSTKREBS Patientenratgeber zu den AGO-Empfehlungen 2023	Patient companion based on AGO recommendations	Patients	AGO Breast Commission with AGO Patient Forum.
Voiß P. (2018) Möglichkeiten und Grenzen der Komplementärmedizin.	Information leaflet	Patients	Brustkrebs Deutschland e.V.

### Model Preparation

We used OpenAI’s feature to build custom GPTs, based on GPT-4, with user-defined instructions and access to a document database for RAG [37,38]. The retrieval mechanism can find relevant information from the uploaded documents and pass it along with the original question to the GPT. For our experiments, it was sufficient to upload the documents one by one. All technical details (eg, splitting documents into chunks, embedding chunks to obtain a vector index, question embedding, and similarity-based matching) are handled by OpenAI. The RAG mechanism is used as is, since parameters that affect retrieval (eg, threshold values for similarity measures or number of retrieved documents) are not exposed. A detailed explanation of the RAG technology can be found elsewhere [33,39,40].

We experimented with different instructions in the system prompt using a set of 5 short questions (included in Multimedia Appendix 1) that the authors formulated before receiving test questions from the Patient Representation Group. Based on the initial experiments, we agreed on using the following five instructions: (1) search for relevant information in the documents uploaded; (2) clearly advise against therapies that are not evidence-based; (3) ask clarifying questions, if necessary; (4) formulate empathetic answers; and (5) not to mention severe complications unless they are clearly indicated by the patient; implemented through the system prompt (Figure 1B). Since our goal is to evaluate the answering capability in the German language, we used the instructions in German (also included in Multimedia Appendix 2). All evaluations took place on or before April 19, 2024.

### Test Questions

We asked one of Germany’s largest BC Patient Representation Groups to share a set of commonly asked questions. We indicated that we have a focus on medical questions only and made no further recommendations as to topic, number, difficulty, or length of the question. Questions were submitted to the Patient Representation Group in person at regional or national meetings, via phone, email, or various online social network platforms that the BC Patient Representation Group is active on. The Patient Representation Group selected the questions based on their judgment of their importance and frequency of occurrence in real-world patient interactions. They grouped the questions into 7 categories (Aftercare, Bone health, DCIS [ductal carcinoma in situ], Diagnostics, Diet and nutritional supplements, Complementary medicine, and Therapy). Answers were not provided.

We performed an initial screening of each received question to determine whether its nature is medical and excluding legal and reimbursement questions. Exclusion decisions are based on consensus between 2 reviewers (SB and UM). All questions (included and excluded) are included in the Multimedia Appendix 1.

Most remaining questions are used in the evaluation “as-is,” even if the question is complex or with a high potential of misinterpretation. We edited some questions by writing out abbreviations or adding the category at the start of the question. The edits are detailed in Table 2.

**Table 2.** Edits on the original questions before using them to evaluate our chatbot.

Original	Modification	Translation	Affected questions
AI^a^	Aromatase-inhibitor	Aromatase inhibitor	17, 34, 37, 38, 41, 78, and 104.
Empty	Category placed in front of the question for context (eg, Brustkrebs, hormoneller Brustkrebs, Ernährung und Nahrungsergänzungsmittel, Hitzewallungen und Schweißausbrüche (vasomotorische Symptome))	Breast cancer, hormonal breast cancer, nutrition and dietary supplements, excessive sweating (vasomotor symptoms)	74, 75, 76, 77, 89, 96, and 98.
NEM	Nahrungsergänzungsmittel	Dietary supplements	76, 80, 82, 88, and 102.

a AI: aromatase inhibitor.

### Evaluation

Evaluating the output of LLMs in medical question-answering systems is a topic of current debate, with consortia developing standards [41]. To date, a standardized evaluation framework is missing. Typically, evaluation criteria are defined at the outset of the study and evaluated on a Likert scale [42-45].

We consented to 4 criteria on which to evaluate the chatbot after feedback from the Patient Representation Group. It was agreed that the Patient Representation Group rate the comprehensibility of the answers (“The answer is clear to me.”), a senior BC expert (ML), who is represented in several German guideline commissions and on the board of the German Society for Senology, rated correctness (“The answer presents scientifically correct information.”), completeness (“The answer includes all important aspects.”) and whether the answer has potential to cause undue harm (“The answer does not cause undue harm.”; Figure 1C). For the Patient Representation Group, 5 raters were recruited by the spokesperson during an in-person event of regional leaders. Each rater independently completed the evaluation using the 5-point Likert scale and a “don’t know / can’t answer” option. Only aggregate response counts per item were collected; no individual-level data were recorded. Direct interaction with individual patient raters was not pursued due to legal and ethical considerations.

The 104 questions are posed to the LLM once. To rate an answer, the raters evaluated each statement on a 5-point Likert scale (5=Strongly agree, 4=Agree, 3=Neutral, 2=Disagree, and 1=Strongly disagree). For each of the given criteria, we present individual ratings per category divided by the total number of ratings for the given category.

### Ethics Statement

According to §15 of the Professional Code of the Berlin Medical Association, research based solely on anonymized data is exempt from the requirement for formal ethical approval. In line with this provision, we did not obtain ethics committee approval for this study, as only aggregated, deidentified data were used. However, we recognize that the Ethics Committee’s guidance encourages researchers to seek consultation even when data are deidentified or aggregated. Prospective consultation with the ethics committee was not sought.

Participants were invited to complete a paper-based form, and there was no direct interaction between the research team and patients. All communication and data collection were managed by a spokesperson from the Patient Representation Group, who aggregated and anonymized the responses before sharing them with the research team. All data were handled in accordance with applicable data protection regulations and shared in anonymized, aggregated form only.

No direct compensation was provided to individual participants. A modest compensation was provided to the Patient Representation Group for their coordination efforts and data aggregation in accordance with the FSA Code for Collaboration with Patient Organizations specified in the EFPIA Code of Practice (2008). All financial contributions and contracts with Patient Representation Groups are publicly disclosed in the “Transparenzkodex” annually.

## Results

### Questions

**Figure 2.**
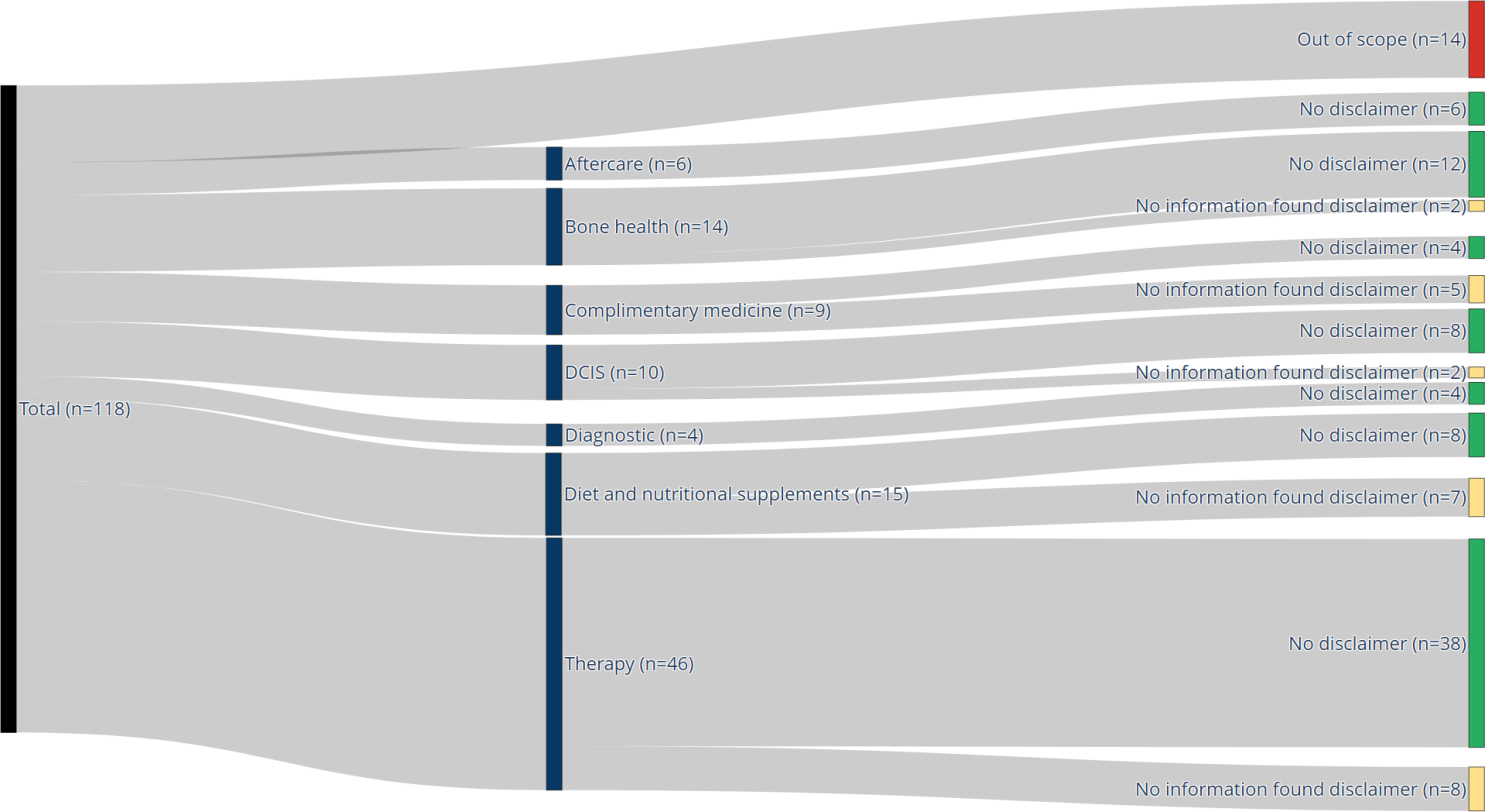
In total, we received 118 questions (black). Human review determined that 14 questions are out of scope (red). The remaining questions are grouped into different categories (blue). Color codes green and yellow indicate whether the model retrieves relevant information from the documents. DCIS: Ductal carcinoma in situ.

We received a total of 118 questions and a corresponding category for each question. A total of 14 questions are excluded because they were related to nonmedical aspects (eg, insurance coverage, reimbursements, and legal aspects). The questions are grouped into 7 categories (Aftercare, Bone health, DCIS, Diagnostics, Diet and nutritional supplements, Complementary medicine, and Therapy). Figure 2 shows the breakdown of the number of questions per category. Many questions were asked on managing side effects, particularly for endocrine and endocrine-based therapy, with a specific emphasis on complementary and alternative medicine, as well as dietary supplements.

For some questions, the LLM reported that no relevant information was found in the documents included. This means that the retriever mechanism was not able to match a query to information stored in the adjacent database. In several cases, the RAG-based model returned no relevant documents from the retrieval component. As internet search was not enabled in our experimental setup, the LLM proceeded to generate responses based solely on its pretrained and fine-tuned knowledge. These instances are reported as part of the results, reflecting the system’s behavior when retrieval fails. The model reported for 24 questions that no relevant information was found (see Figure 2).

### Chatbot Answers and Evaluation

The answers were evaluated using the four criteria (1) correctness, (2) completeness, (3) no harm, and (4) comprehensibility, either by a senior BC expert (criteria 1‐3) or the Patient Representation Group (criteria 4). For each criterion, we formulated a statement (see the Methods section) and evaluated the statement along a 5-point Likert scale. By combining “Strongly Agree” with “Agree” and “Disagree” with “Strongly Disagree,” we see that:

- In total, 427 out of 499 (85.6%) ratings of the answers are rated as comprehensible and 42 (8.4%) as incomprehensible.
- A total of 91 out of 104 (87.5%) answers are rated as correct and 7 (6.7%) incorrect.
- In addition, 72 out of 104 (69.2%) answers are rated as including all relevant information, and 20 (19.2%) have some missing information.
- Furthermore, 93 out of 104 (89.4%) answers are rated as not harmful, and 6 (5.7%) may cause potential harm.

The “Neutral” ratings account for the remaining percentages up to 100. Note that comprehensibility was rated by multiple raters, hence the number of ratings exceeds the number of questions.

A total of 6 answers are considered potentially harmful. One answer related to gene expression testing was classified as potentially harmful due to an incorrect statement on reimbursement. In addition, 2 answers on the use of abemaciclib did not provide information on its use in premenopausal women or failed to mention the absence of data regarding the initiation of abemaciclib therapy 2‐3 years after starting endocrine therapy. Furthermore, 2 answers were deemed potentially harmful because they provided individualized recommendations for the duration of aftercare, including breast sonography. Finally, the chatbot mentioned hormone replacement therapy as a treatment for vasomotor symptoms but failed to mention nondrug therapies.

A total of 2 answers were deemed incomplete due to missing reimbursement information. In addition, some answers failed to mention new therapeutic treatment regimens, lacked comprehensive details on aftercare or testing, or omitted aspects related to endocrine therapy, osteo-oncology, or hormonal testing for menopause status.

**Figure 3.**
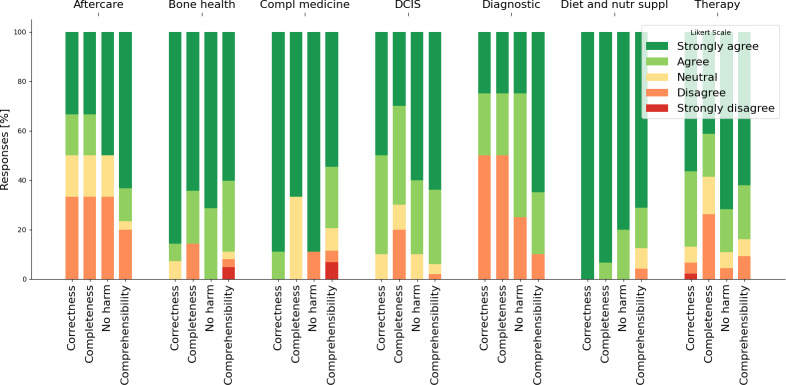
Evaluation of the 4 criteria (Comprehensibility, Correctness, Completeness, and No harm) using a 5-point Likert scale for each question category. Compl: Complimentary; DCIS: Ductal carcinoma in situ; Nutr suppl: nutrition supplement.

We analyze the answers for each category separately (see Figure 3 and Table 3). We observe that the chatbot performs well across all criteria for Bone health (14 questions) and diet and nutritional supplements (15 questions). For the latter category, the chatbot reported no relevant information found in the documents for 7 out of 15 questions (see Figure 2). The worst-performing categories (Aftercare and Diagnostics) also have the fewest number of questions (6 and 4, respectively). Some answers (20 out of 104) omit important information, except for the Diet and nutritional supplement category. A total of 6 responses were identified as potentially harmful by expert judgment, typically for omitting important caveats about medication use or not mentioning alternative (nondrug-based) therapies. One answer on abemaciclib therapy did not include information on its use in premenopausal women. Another answer failed to mention the absence of data regarding the initiation of abemaciclib therapy 2‐3 years after starting endocrine therapy. Furthermore, 2 answers were deemed potentially harmful because they provided individualized recommendations for the duration and type of aftercare.

**Table 3.** Individual ratings per category along the 4 dimensions.

Category	Questions	No information found	Dimension	Ratings, n				
				5	4	3	2	1
Aftercare	6	0	Correctness	2	1	1	2	0
			Completeness	2	1	1	2	0
			No harm	3	0	1	2	0
			Comprehensibility	19	4	1	6	0
Bone health	14	2	Correctness	12	1	1	0	0
			Completeness	9	3	0	2	0
			No harm	10	4	0	0	0
			Comprehensibility	38	18	2	2	3
Complementary medicine	9	5	Correctness	8	1	0	0	0
			Completeness	6	0	3	0	0
			No Harm	8	0	0	1	0
			Comprehensibility	24	11	4	2	3
Ductal carcinoma in situ	10	2	Correctness	5	4	1	0	0
			Completeness	3	4	1	2	0
			No harm	6	3	1	0	0
			Comprehensibility	32	15	2	1	0
Diagnostic	4	0	Correctness	1	1	0	2	0
			Completeness	1	1	0	2	0
			No harm	1	2	0	2	0
			Comprehensibility	13	5	0	2	0
Diet and nutritional supplements	15	7	Correctness	15	0	0	0	0
			Completeness	14	1	0	0	0
			No harm	12	3	0	0	0
			Comprehensibility	52	12	6	3	0
Therapy	46	8	Correctness	26	14	3	2	1
			Completeness	19	8	7	12	0
			No harm	33	8	3	2	0
			Comprehensibility	136	48	15	20	0

Finally, the chatbot mentioned hormone replacement therapy as a treatment for vasomotor symptoms (as side effects of endocrine therapy). However, hormone replacement therapy is a contraindication for breast cancer patients in this specific therapeutic situation (Table 3).

## Discussion

### Principal Findings

One of the largest German BC Patient Representation Group shared a set of typical questions (without identifiable information) that commonly arise. These questions highlight significant and specific information needs of patients—specially regarding endocrine or endocrine-based therapy and the prevention or management of side effects—that go beyond the scope of general health literacy support. Unlike other AI or chatbot or patient vignette studies, this BC Patient Representation Group provided relatively complex medical questions in German, not easily answered through a simple Google search or by referring to patient versions of guidelines.

In response, we evaluated whether a RAG and LLM-based chatbot solution could provide high-quality, tailored information in German. The database included authoritative German-language guidelines and patient information materials that are valid for Germany. Overall, the answers to 104 BC questions were evaluated on 4 criteria (comprehensibility, correctness, completeness, and potential harm) using a 5-point Likert scale. Despite an overall high quality of responses, 20 out of 104 answers were incomplete, and 6 were potentially harmful.

In summary, the chatbot was tested on 104 frequently asked breast cancer–related questions and produced answers that were mostly rated positively across 4 criteria (comprehensibility, correctness, completeness, and harm potential). Specifically, 85.6% (427 out of 499 ratings) of responses were deemed comprehensible, 87.5% (91/104) correct, and 89.4% (93/104) free of undue harm; however, only 69.2% (72/104) of answers were judged complete. Shortcomings primarily involved incomplete details on reimbursement or newer treatment regimens and omissions regarding aftercare or endocrine therapy.

Potentially harmful guidance stemmed mainly from two issues: (1) outdated or ambiguous source material (eg, reimbursement rules that had changed, guidance on hormone replacement therapy lacking caveats about medication use, or not mentioning alternatives) and (2) retrieval gaps that prevented the model from grounding its answer in the most relevant passages. When the necessary nuance was scattered across tables, figures, or inconsistent terminology (“aftercare,” “follow-up,” and “screening”), the retrieval component sometimes surfaced a partial context, prompting the LLM to fill the vacuum with general knowledge that did not fit the oncological edge cases.

For example, the answer related to gene expression testing was classified as potentially harmful due to an outdated statement on reimbursement found in the source document [46]. In addition, no context was provided for the use of abemaciclib on premenopausal women, likely because the 2018 and 2021 guidelines [47,48] did not provide data on its use in early breast cancer.

The chatbot may have been misled for the recommendation of individualized recommendations for the duration and type of aftercare by the varying terminologies used in the documents (“aftercare,” “follow-up,” and “screening”), different design formats, tables, and inconsistent time formats (months, years; in numbers or text). In addition, considerations for specific situations such as DCIS, breast scarring after surgery, or the statement that aftercare can be adapted to the symptoms may have contributed to the “confusion.” While potentially harmful answers were identified through expert review, actual harm to patients remains unlikely due to clinical safeguards.

Notably, the chatbot performed better than expected in the areas of bone health and nutrition, even when relevant documents were not retrieved, suggesting that its base training data sufficed to answer many diet-related questions. Overall, the system demonstrated promising accuracy and clarity but showed room for improvement in providing comprehensive and fully risk-aware medical guidance.

Several questions encompassed multiple aspects and included subquestions that required broad answers to cover all points (see Multimedia Appendix 1). Incomplete answers were primarily due to the absence of reimbursement details, newer therapeutic regimens not yet documented in guidelines, and retrieval limitations affecting tabular and graphical data representation. In 3 cases, there was a lack of clinical data to address the specific question. For 16 other cases, there was an absence of information in the source documents. Specifically, in 2 of these cases, information on new therapeutic treatment regimens was not yet included in any guidelines. In another case, outdated guideline information led to an inaccurate and incomplete answer. In addition, in 2 cases, the topic of reimbursement was inappropriately covered, despite it typically being out of scope for the source documents. Furthermore, in 8 instances, the available information was not retrieved, probably because it was presented in tables, figures, or listings with ratings (such as on level of evidence and on the grade of recommendation) rather than text, making it accessible only to medical experts who could interpret it, a task beyond the capability of the current retrieval mechanism. The inconsistent terminologies and varying design and time formats across and within the source documents might have also contributed to the nonretrieval of information.

The curation of up-to-date and machine-accessible information in the database improves results. We hypothesize that a textual representation of information from tables, figures, or listings improves results with the given RAG technology. Alternatively, digitized and annotated versions of relevant documents [49] or multimodal RAGs [50] likely improve retrieval.

To mitigate the risk of potentially harmful or incomplete answers, it is important to provide a disclaimer that the chatbot is not able to replace consultation with medical professionals and encourage patients to seek consultation. We strongly suggest that continuous monitoring in the form of transcript reviews and human oversight by medical professionals is implemented, as others have pointed out as well [44]. Furthermore, it is clear that chatbots are classified as medical devices [31] and need substantial safety measures before being used at scale.

### Comparison With Previous Work

In agreement with our results, it has been observed that ChatGPT scores high on correctness and often significantly lower on completeness for cancer-related question-answer pairs in an English-language setup [51,52]. A growing body of literature demonstrates that the RAG mechanism helps to ground LLMs in factual information for medical use cases [53,54]. To the best of our knowledge, there is no similar study evaluating LLM-based chatbots on German question-answer pairs for any cancer entity. The training on English-language data may introduce subtle biases from the respective health care systems. Our results show that ChatGPT, together with authoritative documents, can answer BC questions in German with similar performance.

Our realistic set of real-world patient questions can be used by other researchers to develop German BC chatbots. Many questions raised by the BC Patient Representation Group pertain to endocrine and endocrine-based therapy, particularly related to the prevention or management of side effects. Providing active patient support for this therapy is crucial to ensure adherence and prevent early termination, as discontinuation of endocrine or endocrine-based therapy is associated with worse outcomes, including reduced overall survival [55].

A further topic of interest among patients is food supplements and alternative medicine. Evidence-based recommendations on complementary and alternative medicine can help patients avoid negative interactions with cancer treatment, prevent harmful or ineffective therapies, and potentially contribute to treatment success [56]. The chatbot might provide substantial support for patients, especially when based on evidence-based information such as from the German S3 guidelines on complementary medicine in oncology.

This evaluation of a chatbot prototype in German language provides a strong baseline for evaluating German BC chatbot apps. We observe that ChatGPT, together with appropriate documents in the vector store, provides a strong performance even for a German medical question answering task.

### Limitations

First, we pose each question to the chatbot only once. Thus, we do not assess whether the retrieval mechanism consistently retrieves the same result or if the LLM’s answers are consistent across repeated queries.

Second, the evaluation criteria—correctness, completeness, and potential for undue harm—are only evaluated by a single expert, introducing subjective judgment and potential bias representing a subjective evaluation.

Third, it is possible that the Patient Representation Group introduces a bias in the question selection.

Fourth, our study does not simulate real-world interactions, where patients typically ask follow-up questions and discuss outcomes directly with their treating physicians. A comprehensive real-world evaluation would require extended and interactive dialogues between patients and the chatbot.

Fifth, although we share test questions, source documents and prompts, reproducibility is only partially achievable because our study relies on closed-source algorithms for information retrieval and output generation by the LLM, whose parameters and implementations may change over time.

### Future Directions

Since we observe that some questions are not directly addressed in the included documents, a future model can be improved by including relevant journal articles or guidelines and recommendations from other countries. It is well-known that the prompting strategy can influence model performance [57]; therefore, improved prompting (eg, chain-of-thought [58], multiround iterative questioning [59], or self-reasoning [60]) may significantly improve results. In addition, specific fine-tuning on a BC question answering task can be expected to improve performance [61].

A total of 14 questions revolved around insurance coverage, reimbursement, and legal aspects of social law, which underscores the need for specific support in these areas. For our study, we consider these topics out of scope, since an interdisciplinary collaboration between legal and social service experts would be required and a different set of authoritative documents. However, it is crucial to address these issues, as a cancer diagnosis poses a high risk of financial problems for patients [55]. An enhanced chatbot or a chatbot specifically designed to address patient questions in these areas would increase.

### Conclusion

A cancer diagnosis is a major turning point in life for most, as it is potentially life-threatening and life-changing. We evaluate an AI-based chatbot in answering realistic and challenging patient questions in the German language. The BC chatbot prototype provides largely accurate, comprehensible, and safe answers for German-speaking patients with BC, but incomplete information remains a limitation, particularly concerning reimbursement and newer treatments. The technology may provide real value today, as it can always be used easily and might help to meet patients’ needs for information. Further development, testing, and evaluation of chatbots for patients is a multidisciplinary endeavor that should involve patients actively in this process. In addition, the inclusion of guardrails and prominent disclaimers that technology cannot replace professional consultation and human oversight is important for apps.

## Supplementary material

10.2196/68426 Multimedia Appendix 1 Questions for initial experimentation, prompts, and incomplete answers.

10.2196/68426 Multimedia Appendix 2 Questions of the Patient Representation Group along with answers by the chatbot.

10.2196/68426 Checklist 1 TRIPOD+LLM (Transparent Reporting of a Multivariable Prediction Model for Individual Prognosis or Diagnosis+Large Language Model) checklist.

## Abbreviations

AGO: Arbeitsgemeinschaft Gynäkologische Onkologie e.V.
AI: artificial intelligence
BC: breast cancer
DCIS: ductal carcinoma in situ
GPT: Generative pretrained transformer
HCP: health care professional
LLM: large language model
RAG: retrieval-augmented generation
TRIPOD+LLM: Transparent Reporting of a Multivariable Prediction Model for Individual Prognosis or Diagnosis+Large Language Model
