# Supplementary material for: Evaluating a Chatbot as a Companion for Patients With Breast Cancer: Collaborative Pilot Study
Source: JMIR Cancer. 2025 Aug 13;11:e68426. doi: 10.2196/68426 (PMC12373300; doi:10.2196/68426)
Supplement: Multimedia Appendix 1 [file cancer-v11-e68426-s001.docx]

## Appendix

### Questions for initial experiments

Prior to receiving test questions from the Patient Representation Group, we experimented with the chatbot using the following 5 hypothetical patient questions:

1. “Mein Tumor ist zu groß und kann nicht operiert werden. Muss ich wirklich eine Chemotherapie machen?“
2. „Nach der Chemotherapie ist mein Tumor verschwunden. Ist die OP wirklich noch notwendig“?
3. „Mein Brustkrebs wurde entfernt. Muss ich jetzt wirklich noch eine Chemotherapie machen? Oder geht es auch ohne?“
4. „Wie lange werde ich bei meinen Metastasen durch meinen Brustkrebs noch leben?“
5. „Welche sanften Heilmethoden und Alternativen zur Schulmedizin kann ich bei meinem Tumor in der Brust (ca. 10 cm x 10 cm) anwenden?“

### Instructions

1. Schaut bei jeder Frage in die hochgeladenen Dokumente und sagt wenn zu der Frage keine Informationen gefunden wurden
2. Spricht sich deutlich gegen Therapien aus, die nicht auf Evidenz basieren. Dabei wird klar das „Nein“ genannt
3. Stellt Fragen um möglichst patientenspezifische Antworten geben zu können
4. Gibt empathische, mitfühlende Antworten
5. Gibt keine Hinweise auf schwerwiegende Komplikationen die nicht klar anhand der Informationen der Patientin indiziert sind

### Incomplete answers

| Nr. | No clinical data existing to answer the question. | Information not available in source documents | Information is available but was not retrieved | information was retrieved from a source document with outdated information. | The topic is out of scope of the source documents (such as questions on reimbursement). | Comments |
| --- | --- | --- | --- | --- | --- | --- |
| 1 |  |  |  | x | x | Reimbursement topic not covered correctly. |
| 4 | x | x |  |  |  |  |
| 8 |  | x |  |  | x | Reimbursement topic not covered. |
| 13 |  | x |  |  |  |  |
| 15 |  | x |  |  |  | New data not yet incorporated into guidelines. |
| 16 |  | x |  |  |  | New data not yet incorporated into guidelines. |
| 17 | x | x |  |  |  |  |
| 21 |  | x | x |  |  | No clear statement on topic in body text, can only be indirectly deducted from content. |
| 25 |  |  | x |  |  |  |
| 26 |  | x |  |  |  |  |
| 35 |  | x |  |  |  |  |
| 36 |  | x | x |  |  | No clear statement on topic in body text, can only be indirectly deducted from content. |
| 37 |  | x |  |  |  |  |
| 38 |  | x | x |  |  | No clear statement on topic in body text, can only be indirectly deducted from content. |
| 45 |  | x | x |  |  | No clear statement on topic in body text, can only be indirectly deducted from content. |
| 46 | x | x |  |  |  |  |
| 65 |  | x | x |  |  | No clear statement on topic in body text, can only be indirectly deducted from content. |
| 70 |  | x |  |  |  |  |
| 90 |  |  | x |  |  | No consistent terminologies in the source documents, as well as different design formats and time formats. |
| 91 |  |  | x |  |  | No consistent terminologies in the source documents, as well as different design formats and time formats. |
